# Immediate Postpartum Insertion of Copper Intrauterine Device in a Brazilian University Hospital: Expulsion and Continuation Rates

**DOI:** 10.1055/s-0042-1759628

**Published:** 2023-03-06

**Authors:** Georgia Nahas, Claudia Magalhães, Flavia Bueloni-Dias, Eliana Nahas, Vera Borges

**Affiliations:** 1 Faculdade de Medicina de Botucatu, Universidade Estadual Paulista “Júlio de Mesquita Filho”, Botucatu, SP, Brazil

**Keywords:** postpartum contraception, cooper intrauterine device, immediate postpartum insertion, expulsion rate, continuation rate, contracepção pós-parto, dispositivo intrauterino de cobre, inserção no pós-parto imediato, taxa de expulsão, taxa de continuação

## Abstract

**Objective** To evaluate the expulsion and continuation rates of the copper intrauterine device (IUD) inserted in the immediate postpartum period in a Brazilian public university hospital.

**Materials and Methods** In the present cohort study, we included women who received immediate postpartum IUD at vaginal delivery or cesarean s March 2018 to December 2019. Clinical data and the findings of transvaginal ultrasound (US) scans performed 6-weeks postpartum were collected. The expulsion and continuation rates were assessed 6-months postpartum using data from the electronic medical records or by telephone contact. The primary outcome was the proportion of IUDs expelled at 6 months. For the statistical analysis, we used the Student *t* -test, the Poisson distribution, and the Chi-squared test.

**Results** There were 3,728 births in the period, and 352 IUD insertions were performed, totaling a rate of 9.4%. At 6 weeks postpartum, the IUD was properly positioned in 65.1% of the cases, in 10.8% there was partial expulsion, and in 8.5% it had been completely expelled. At 6 months postpartum, information was obtained from 234 women, 74.4% of whom used IUD, with an overall expulsion rate of 25.6%. The expulsion rate was higher after vaginal delivery when compared with cesarean section (68.4% versus 31.6% respectively; *p* = 0.031). There were no differences in terms of age, parity, gestational age, final body mass index, and newborn weight.

**Conclusion** Despite the low insertion rate of copper IUDs in the postpartum period and a higher expulsion rate, the rate of long-term continuation of intrauterine contraception was high, indicating that it is a useful intervention to prevent unwanted pregnancies and to reduce short-interval birth.

## Resumo

**Objetivo** Avaliar as taxas de expulsão e continuação do dispositivo intrauterino (DIU) de cobre inserido no pós-parto imediato em um hospital universitário brasileiro.

**Materiais e Métodos** Neste estudo de corte transversal, foram incluídas parturientes submetidas à inserção de DIU de cobre no pós-parto imediato entre março de 2018 e dezembro de 2019. Foram coletados dados clínicos e da ultrassonografia (US) transvaginal realizada após seis semanas. As taxas de expulsão e de continuação foram avaliadas após seis meses por meio de dados do prontuário ou por contato telefônico. O resultado principal foi a proporção de DIUs expelidos em seis meses. Para análise estatística, utilizaram-se o teste *t* de Student, a distribuição de Poisson, e o teste do Qui quadrado.

**Resultados** Houve 3,728 nascimentos no período, e foram inseridos 352 DIUs, em uma taxa de 9,4%. Com 6 semanas, o DIU estava bem posicionado em 65,1% dos casos, em 10,8%, houve expulsão parcial, e, em 8,5%, fora totalmente expelido. Aos 6 meses de pós-parto, foram obtidas informações de 234 mulheres, 74,4% das quais usavam DIU, com uma taxa de expulsão geral de 25,6%. A taxa de expulsão foi maior após o parto vaginal do que após cesariana (68,4% *versus* 31,6%, respectivamente; *p* = 0,031). Não houve diferenças quanto à idade, paridade, idade gestacional, índice de massa corpórea final, e peso do recém-nascido.

**Conclusão** Apesar da baixa taxa de inserção e alta taxa de expulsão, a taxa de continuação em longo prazo da contracepção intrauterina com DIU de cobre foi elevada, o que indica que se trata de intervenção útil para prevenir gestações indesejadas em curto intervalo de tempo.

## Introduction

The rates of unplanned pregnancies are high, corresponding to ∼ 50% of all pregnancies. 1 To reduce the number of unintended pregnancies and short interpregnancy interval, clinicians should be offering the most effective methods of contraception to women as first-line options. 2 3 Long-acting reversible contraception (LARC) methods are the most effective, as they do not rely on patient compliance and are not user-dependent. 4 Thus, increasing access to LARC, including the copper intrauterine device (IUD), is a critical strategy to decrease the rate of unplanned pregnancies. 1 5

The copper IUD has been widely available as an interval contraceptive method for years, and it has the advantage of being cost-effective and nonhormonal. 1 The estimated pregnancy rate for the typical use of the copper IUD is extremely low, lower than 1 pregnancy per 100 women in the first year of use. 6 The International Federation of Gynecology and Obstetrics (Fédération Internationale de Gynécologie et d'Obstétrique, FIGO, in French) recognized the potential public health impact that the postpartum IUD could have, particularly for women in low- and middle-income countries. 7 The immediate postpartum is a critical moment for contraceptive access and an opportunity to initiate LARC, which includes the insertion of an IUD. 8 9 Provision of immediate postpartum IUD insertion within maternity settings can overcome many of the barriers faced by women in accessing this method after childbirth. The uptake of IUD can help reduce the risk of a subsequent unintended pregnancy and improve spacing between births. 2 10 11

Recent studies 9 12 13 14 have evaluated aspects related to the postpartum insertion of copper IUD, such as efficacy, acceptance, and complications with the use like the expulsion rate for example. The expulsion rates vary widely across studies, ranging from 2% to 27% after vaginal delivery, and from 0% to 20% after cesarean section, without clear evidence about the factors that may influence expulsion. 5 On the other hand, in view of the available scientific evidence, the postpartum insertion of an IUD is a safe practice with few contraindications and many benefits that can be routinely offered to women, including adolescents. 8 9 However, few studies 15 16 17 18 19 have evaluated the results of this practice in Brazilian public maternities. Thus, our objective was to evaluate the expulsion and continuation rates of copper IUDs inserted in the immediate postpartum period in a Brazilian public university hospital.

## Materials and Methods

The present was a prospective cohort study conducted between March 2018 and December 2019 at a Brazilian public university hospital. During the study period, the women were informed about the availability of the IUD in the postpartum. Intentional sampling was used. All women who expressed a desire and received immediate postpartum copper IUD at delivery were enrolled. Adherence to the IUD was voluntary and preceded by a thorough explanation of the method, which was recorded in writing in a medical record. The card accompanying the copper IUD was signed and contained all information about the method, and a copy of this explanatory card was given to the patient. The exclusion criteria for insertion of the IUD were: chorioamnionitis or fever over the previous 48 hours; 2 instances of rupture of membranes > 24 hours in vaginal delivery or > 12 hours in cesarean section; gestational age < 32weeks at the time of delivery; postpartum hemorrhage, uterine atony; extensive vaginal laceration; obstetric diseases with increased risk of coagulation disorders; signs of vaginitis or cervicitis on gynecological examination; anatomic abnormalities that distort the cavity; and personal history of coagulation disorders or immunosuppression. Written informed consent was obtained from all participants, and the study was approved by the Ethics Committee of the Botucatu Medical School, Universidade Estadual Paulista “Júlio de Mesquita Filho” (under CAAE: 15699319.4.0000.5411).

The copper IUD used in all patients (model TCu380A-FURP, Fundação Para o Remédio Popular [FURP], Guarulhos, SP, Brazil) was inserted in the immediate postpartum period (10 minutes after placental delivery) by the medical residents or staff on duty, who had received a brief training provided by the researchers. In patients undergoing cesarean section, after placental delivery, the IUD was manually inserted at the top of the uterine fundus through the hysterotomy incision; then, the strings were directed to the cervix, without cutting them. 15

In vaginal delivery, the IUD was inserted after delivery, with the uterus spontaneously contracted and before the administration of oxytocin. For the vaginal insertion, a 33-cm curved Kelly forceps was used. After placental delivery, the anterior lip of the cervix was clamped with a Collin forceps to straighten the uterocervical canal. The IUD is removed from its prepackaged inserter and advanced into the uterine cavity using a Kelly forceps. After reaching the uterine fundus (confirmed by external palpation with the non-dominant hand), the device is released from the forceps, which is then slowly removed. As the forceps is being removed, a hand rotation of ∼ 15° is performed to prevent IUD exteriorization. 10 After vaginal insertion, the strings of the IUD should not be seen upon inspection of the cervix.

The following clinical data were obtained from the patient records: age, marital status, parity, type of delivery, weight, height, body mass index (BMI), gestational age at delivery, and weight of the newborn. At hospital discharge, all participants were instructed to return for follow-up six weeks after delivery for transvaginal ultrasound and assessment of IUD adaptation and possible complications. Complete expulsion was defined as a device that had been fully expelled from the uterine cavity before the follow-up (self-reported), and partial expulsion was defined as a device found to be located within the cervical canal (either whole or in part) on clinical examination or ultrasound at follow-up, and these were removed. 10

Ultrasound findings were reported as IUD considered properly positioned at the 6-week (inside the uterine cavity, above the internal cervical orifice), completely expelled (empty uterine cavity), and partial expulsion (located in the cervical canal). In the latter situation, the IUD was immediately extracted. In cases in which no device was observed on ultrasound, an abdominal/pelvic X-ray was performed to exclude uterine perforation. Infection was recorded if there was a self-reported or documented history of receiving antibiotics (and/or device removal) for suspected intrauterine/pelvic infection at or before the follow-up. Ultrasound evaluation was performed with an equipment with three-dimensional (3D) technology (Voluson E6, GE Healthcare, Chicago, Il, US) using a convex 6–8-Mhz volumetric transducer (RAB, GE Healthcare). All exams were performed by the same investigator, who was previously trained for them.

The primary outcome was the proportion of IUDs expelled at 6 months, and we also compared the expulsion rates after vaginal birth and after cesarean delivery. For patients whose data on the follow-up regarding IUD use were not included in the medical records, a subsequent telephone contact was made by the investigator six months after the insertion of the IUD, and a brief telephone survey was applied to obtain self-reported information on the expulsion and continuation of use of the IUD.

For the primary outcome of IUD expulsion six months postpartum, the participants were divided into groups of women who experienced and did not experience expulsion. The quantitative variables were compared between the groups using the Student *t* -test or Poisson distribution. The Chi-squared test was used to evaluate the associations involving the frequency of the categorical variables. Bivariate and multivariate logistic regression analyses (with stepwise variable selection; including those with *p* < 0.05) were performed to confirm factors that were significantly associated with complete IUD expulsion or confounded associations of interest. These variables included: age, number of pregnancies and deliveries (including the present one), weight, height, BMI, mode of delivery, gestational age, and weight of the newborn. The Kaplan-Meier with log-rank test was used to compare the survival curve according to discontinuation by expulsion among women with either vaginal or cesarean delivery. The odds ratios (OR) with the 95% confidence intervals (95%CIs) were estimated. A level of significance of 5% or the corresponding *p* -value was adopted in all tests. The Statistical Analysis System (SAS, SAS Institute, Cary, NC, US) software, version 9.2, was used for the analysis.

## Results

**Fig. 1:**
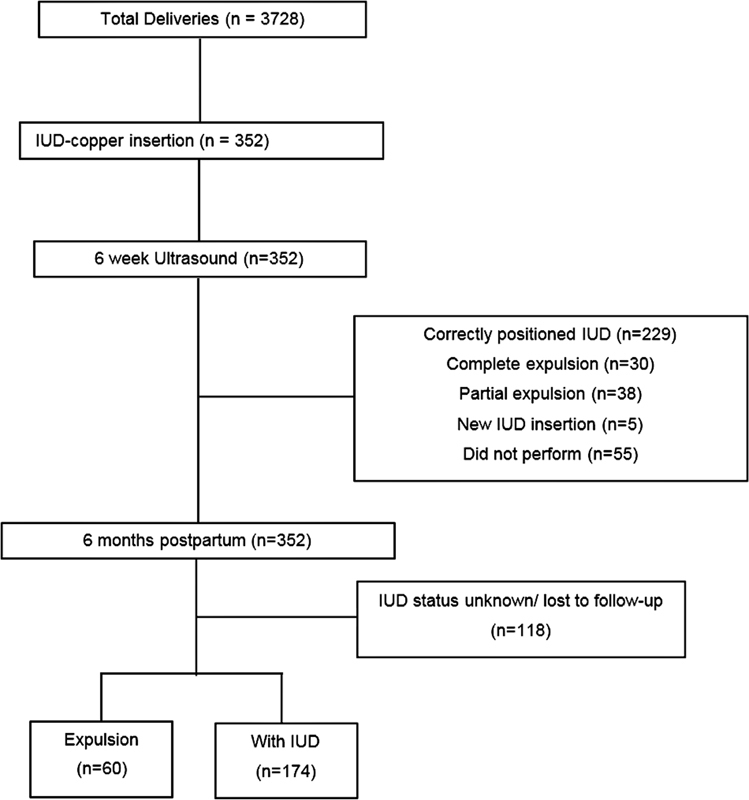
Flowchart of the participants of the present study.

During the study period, 3,728 deliveries occurred in our service, and 352 copper IUDs were inserted, in a rate of 9.4% ( Fig. 1 ). The mean age of the participants was of 28.8 ± 6.2 (range: 15 to 45) years; only 3.7% were under 18 years of age, and 14.5% were primiparous. In 34.1% of the patients the first delivery occurred before the age of 18 years and 26.4% had 4 or more children (average parity 2.8 ± 1.3 children). Most patients (81.2%) reported having a partner, and 53.1%, having a job. Most pregnant women were over 37 weeks of gestational age. At the end of pregnancy, 53.4% of the women were classified as obese (BMI ≥ 30 Kg/m ^2^ ). The insertion of the IUD occurred after vaginal delivery in 53.4%, delivery and during the cesarean section in 46.6% ( Table 1 ).

**Table 1:** Characteristics of the women included in the study ( *n* = 352)

Characteristics	n (%)
Age (years)	
< 18	13 (3.7)
18–29	194 (55.1)
≥ 30	145 (41.2)
Occupation	
Employed	187 (53.1)
No formal employment	165 (46.9)
Relationship status	
Single	66 (18.8)
With partner	286 (81.2)
Age at first delivery (years)	
< 18	102 (34.1)
18–29	186 (62.2)
≥ 30	11 (3.7)
Parity (number of deliveries)	
1	51 (14.5)
2–3	208 (59.1)
≥ 4	93 (26.4)
Body Mass Index at delivery (Kg/m ^2^ ) ^#^	
≤ 24.9	33 (9.4)
25–29.9	89 (25.3)
30–39.9	152 (43.2)
≥ 40	36 (10.2)
Gestational age at delivery	
32 weeks 0 day–36 weeks 6 days	57 (16.2)
37 weeks 0 day–40 weeks 6 days	271 (77.0)
≥ 41 weeks 0 day	24 (6.8)
Type of delivery	
Vaginal	188 (53.4)
Cesarean section	164 (46.6)

Note: ^#^ Data missing for 42 women.

In total, 84.4% (297/352) of the sample returned for the six-week transvaginal ultrasound after delivery. The IUD was properly positioned (in situ) in 65.1% of the cases (229/352). In 10.8% (38/352), there was partial expulsion, and, in 8.5% (30/352), it had been completely expelled. After contraceptive counseling, only 5 of the women chose to undergo a new IUD insertion ( Fig. 1 ). The mean expulsion time in the puerperium period was of 19.1 ± 11.2 days. After 6 months of postpartum, information was obtained from 234 patients (66.5%). Among these women, 74.4% continued using the IUD at 6 months. The overall IUD expulsion rate was of 25.6%, with an average expulsion time of 4.4 ± 1.9 months ( Fig. 1 ).

**Fig. 2:**
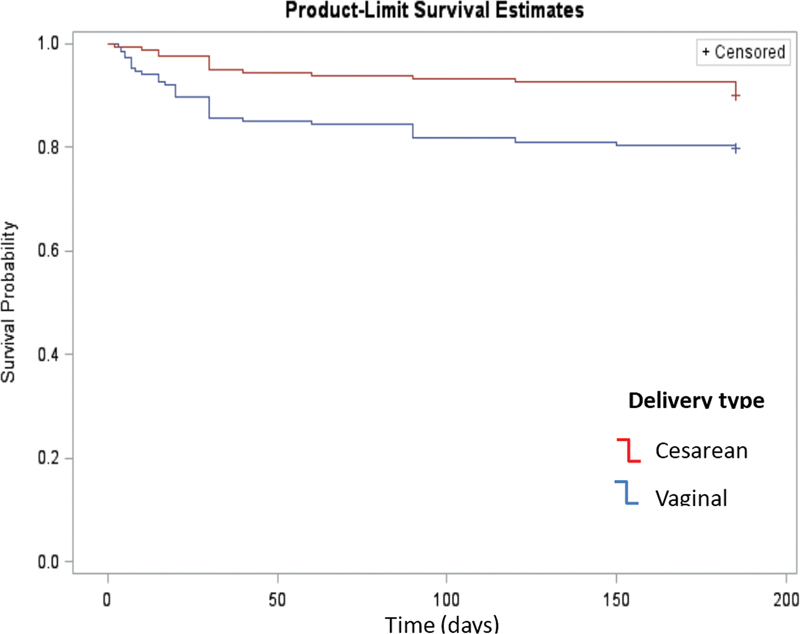
Survival curve (Kaplan-Meier) according to the mode of delivery. Note: *Significance between vaginal and cesarean. Log-rank (Mantel Cox) test: Chi-squared (χ ^2^ ) = 7.64; LF = 1; *p* = 0.006.

Table 2 shows the statistical comparison between women who remained with the IUD at the six-month follow-up ( *n* = 174) and those who experienced expulsion ( *n* = 60). A higher frequency of vaginal delivery was observed among women who experienced expulsion when compared with those who did not (68.4% versus 52.3% respectively; *p* = 0.031). The expulsion rate among those submitted to cesarean section was of 31.6%. There was no difference between the groups regarding age, parity, gestational age at delivery, BMI, and weight of the newborn ( Table 2 ). Fig. 2 shows the survival curve (Kaplan-Meier) according to mode of delivery. During the follow-up, there was greater discontinuation due to expulsion after vaginal delivery when compared with cesarean section ( *p* = 0.006).

**Table 2:** Comparison between women who remained with the intrauterine device at the six-month follow-up and those who experienced expulsion

Variables	Total ( *n* = 234): n (%)	Without IUD ( *n* = 60): n (%)	With IUD ( *n* = 174): n (%)	*p* -value*
Age (years)				
< 18	3 (1.3)	1 (1.7)	2 (1.1)	0.94
18–29	127 (54.3)	32 (53.3)	95 (54.6)	
≥ 30	104 (44.4)	27 (45.0)	77 (44.2)	
Parity (number of deliveries)				
1	32 (13.6)	6 (10.0)	25 (14.9)	0.53
2–3	141 (60.3)	36 (60.0)	105 (60.3)	
≥ 4	61 (26.1)	18 (30.0)	43 (24.7)	
Delivery (including the present one)				
Primiparous	29 (12.4)	6 (10.0)	23 (13.2)	0.51
Multiparous	205 (87.6)	54 (90.0)	151 (86.8)	
Gestational age (weeks completed)				
32 weeks 0 day–36 weeks 6 days	37 (15.8)	9 (15.0)	28 (16.1)	0.24
37 weeks 0 day–40 weeks 6 days	183 (78.2)	50 (83.3)	133 (76.4)	
≥ 41 weeks 0 day	14 (6.0)	1 (1.7)	13 (7.5)	
Weight of the newborn (Kg)				0.21
< 3.0	68 (36.0)	12 (25.6)	56 (39.6)	
3–4	106 (56.1)	30 (63.8)	76 (53.5)	
≥ 4.0	15 (7.9)	5 (10.6)	10 (7.0)	
Type of delivery				
Vaginal	132 (56.4)	41 (68.4)	91 (52.3)	0.03
Cesarean section	102 (43.6)	19 (31.6)	83 (47.7)	
Body Mass Index at delivery (Kg/m ^2^ ) ^#^				
≤ 24.9	17 (7.3)	4 (8.5)	13 (7.8)	0.99
25–29.9	56 (23.9)	12 (25.5)	44 (26.5)	
30–39.9	119 (50.9)	26 (55.3)	93 (56.0)	
≥ 40	21 (9.0)	5 (10.6)	16 (9.6)	

Notes: ^#^ Data missing for 21 women. *Significant difference if *p* < 0.05 (Chi-squared test).

Multivariate logistic regression modeling demonstrated that the expulsion was higher after vaginal delivery than after cesarean section (OR: 2.03; 95%CI: 1.06–3.90; *p* = 0.042). There was no association between expulsion rate and the other risk factors assessed (age, parity, gestational age, BMI, and weight of the newborn).

## Discussion

In the present study, among the women cared for in public university hospital, we observed that, despite the low insertion rate and higher expulsion rate, the continued use of the IUD six months postpartum was high, revealing the importance of the program to expanding access to copper IUDs in the immediate postpartum period. The copper IUD is recommended by the American College of Obstetricians and Gynecologists (ACOG) as one of the best contraceptive options in the immediate postpartum period to improve the spacing of pregnancy, thus contributing to the improvement of maternal and child health care, especially in developing countries. 20 Making immediate postpartum LARC widely available is a promising public health approach to help women achieve a longer interpregnancy interval. 3

At 6 months postpartum, in the whole cohort, the overall rate of expulsion of IUDs was of 25.6%, and the rate of continued use was of 74.4%. A systematic review ^12^ examined the outcomes of IUD insertion immediately after placenta delivery, especially when compared with insertion at other postpartum times. The rate of expulsion of the IUD in 6 months was more frequent in the group of women with immediate insertion (17%) compared with the insertion of the IUD in the return to the puerperium (3%). However, the use of an IUD was more likely with immediate insertion than with insertion in the puerperium (81% versus 67% respectively). For the authors, 12 the benefit of effective contraception immediately after delivery may outweigh the disadvantage of increased risk of expulsion. Gurney et al. 11 evaluated the proportion of copper IUDs expelled or retained that were inserted after vaginal delivery ( *n* = 162 participants). In the six-month study visit, 55.6% had their original IUD in the correct position and continued to use it for contraception. 11 A recent systematic review and meta-analysis 13 provided updated and more detailed pooled IUD expulsion rates and expulsion risk estimates among women with postpartum IUD placement. Complete expulsion rates varied by the timing of placement as follows: 10.2% for immediate; 13.2% for early inpatient; and 1.8% for interval placements. Compared with interval placement, immediate and early postpartum placements were associated with a greater risk of complete expulsion. 13 More importantly, even with a high expulsion rate, ∼ 70% of women who opt for immediate insertion of the IUD in the postpartum period consider it a highly effective and long-lasting contraceptive method. 9

Postpartum IUD expulsion rates vary by timing of placement, IUD type, and delivery method. 21 In the present study, IUD insertion occurred in 53.4% of the sample after vaginal delivery and in 46.6% at cesarean section. The expulsion rate was higher after vaginal delivery when compared with cesarean section (68.4% versus 31.6% respectively). Similar results were observed in a study 22 involving six low-income countries: Sri Lanka, India, Nepal, Bangladesh, Tanzania, and Kenya. A total of 36.766 IUD insertions were performed. Following cesarean delivery, after adjusting for country, women were 67% less likely to experience expulsion than following insertion after vaginal delivery. 22 In the study by Hochmuller et al., 16 the rate of expulsion of IUDs inserted during the immediate and mediate puerperium ( *n* = 170 women) was of 28.8%, and there was no significant association between the occurrence of expulsion and the timing of IUD insertion (immediate: 26.6%; mediate: 34.78%). However, vaginal delivery was four times more likely to be associated with expulsion of IUDs inserted in the puerperal period than during cesarean section. 16 Laporte et al., 17 evaluating 170 women 3 months postpartum, observed that the expulsion rate was higher among those submitted to vaginal delivery than those submitted to cesarean delivery, and that most of the expulsions occurred within 42 days. 17 There is no clear reason for the lower expulsion rates with IUDs placed during a cesarean delivery compared with vaginal delivery, but this may be related to true fundal IUD position at insertion, to the fact that the uterus is more contracted after cesarean deliveries than within 10 minutes of a vaginal delivery, or to the increased likelihood of a less dilated cervix at the time of delivery. 8

The present study has some limitations. First, its cross-sectional design does not enable the establishment of a cause-effect relationship, and any associations cannot imply causality. Second, our findings are limited by the fact that the participants were only recruited from one center. Third, some data was collected by telephone, thus subjecting it to the risks of recall bias. Fourth, we did not evaluate the healthcare provider experience in IUD insertion. Nevertheless, the strength of the present study is that it reflects the reality of many public maternities located in regions of low socioeconomic status, where IUD insertion in the puerperal period can contribute to better family planning.

## Conclusion

In conclusion, despite the low insertion rate of the copper IUD in the postpartum period and a higher expulsion rate, the rate of continuation of intrauterine contraception was high, indicating that it is a useful intervention to prevent unwanted pregnancies in a short period of time. Through shared decision-making, health professionals can help women clarify their reproductive goals and understand contraceptive options, including copper IUDs. The greater risk of expulsion associated with the immediate insertion of the IUD in the postpartum period must be balanced against the benefits of a sustained, reversible, and highly-effective contraception.
